# Availability, Strengths and Limitations of US State Driver’s License Data for Obesity Research

**DOI:** 10.7759/cureus.518

**Published:** 2016-03-03

**Authors:** Benjamin Littenberg, Derek Lubetkin

**Affiliations:** 1 General Internal Medicine Research, University of Vermont; 2 School of Medicine, Tufts University School of Medicine

**Keywords:** obesity, geography, environment and public health, epidemiological data, geographic information systems, rural health

## Abstract

Objectives: Driver’s license records in the United States typically contain age, sex, height, weight, and home address. By combining the body mass index (calculated from the reported height and weight) and address information, researchers can explore and quantify the relationships between obesity and specific environmental features surrounding the place of residence. We report here our experience obtaining those data and the current state of driver’s license data as an epidemiological resource.

Methods: The specific state agency responsible for maintaining driver’s license databases was contacted by email, phone, or both methods for each of the 50 states and the District of Columbia.

Results: Fourteen states with a combined population of 89.8 million people indicated they could provide a total of 73.3 million unique driver’s license (and non-driver identification) data records with address, height, weight, gender, and age, representing 82% of the population in these states. Four additional states will provide data with a zip code but not the street address. A total of 52.6 million unique analyzable records from seven states has been acquired and analyzed. Obesity is more prevalent among males and those living in less urbanized areas.

Conclusion: Driver’s licenses represent an underused resource for studying the geographic correlates of obesity and other public health issues.

## Introduction

Obesity is one of the nation’s most pressing public health issues [1-2] and is a common topic of epidemiological research [3-6]. Body Mass Index (BMI) is the standard measure used to evaluate obesity and is calculated by dividing the weight in kilograms by the height in meters squared [7-8]. Current research often relies on data aggregated at the county, zip code, or census tract level to study trends in body mass [9]. Previous studies have combined those data with data from geographic information systems (GIS) to examine obesity’s relationship to various areal population characteristics, including the proximity of various types of businesses, public facilities, and institutions such as restaurants, grocery stores, gyms, hospitals, parks, and other recreational amenities [10-11]. However, analyses of aggregated data are unsuitable for detecting very local effects of the environment and limit researchers’ ability to adjust for individual factors that vary within geographic clusters. Because of privacy concerns, address-level data on height and weight are not generally available for research.

Driver’s license databases in the United States offer a potential source of data that not only contain the height and weight measures necessary to calculate BMI, but also provide addresses associated with these individual data points. By combining the BMI and address data derived from driver’s license information with GIS data that include rich detail on the built environment, researchers can explore and quantify the relationships between BMI and specific environmental features with greater granularity and precision [12-13].

Although driver’s license lists do not contain information on all individuals, they cover a very high percentage of people between ages 15 and 64, including many non-drivers who receive identification cards [14]. However, access to driver’s license databases is limited by the 1994 federal Drivers Privacy Protection Act (DPPA), Public Law 103-322 (18 U.S.C. § 2721 to §2725) as well as various state laws and regulations [13]. The DPPA makes the information contained in state driver’s license databases protected information that may only be released following the consent of the individual driver, or if the data request falls under one of the fourteen permissible use categories. Category 5 is especially relevant for research.

Category 5: “*For use in research activities, and for use in producing statistical reports, so long as the personal information is not published, redisclosed, or used to contact individuals.”*

Individual states retain the authority to further restrict access to the information contained within driver’s license data. In practice, the availability of driver’s license data varies from state to state.

A survey of the 50 US states and the District of Columbia in 2009 and 2010 found that 22 states denied access to the data, 16 allowed access, and 12 did not provide definite answers [16]. That study did not actually collect the records of the 75 million licenses that were reported as available.

Our own work on the relationship between the built environment and body mass prompted us to gather as many records from these databases as possible. We report here our experience obtaining those data and the current state of driver’s license data as an epidemiological resource. Our specific aims were to collect as many US driver’s license records as possible, develop methods for analyzing them, estimate the fraction of the US population they represent, and report preliminary descriptions of the data. Subsequent research will examine the relationships between BMI and the built environment.

## Materials and methods

The specific state agency responsible for maintaining driver’s license databases was contacted for each of the 50 US states and the District of Columbia. Initial contact began in November 2013 by telephone and e-mail as directed by the state agency’s website. We described our affiliation with the University of Vermont, the data requested, and the plan to explore the relationship between obesity and the physical environment surrounding the place of residence. We specifically requested that the state provide a data file containing drivers’ age (or year of birth), sex, height, weight, date of issue, date of expiration, and address for all drivers in their state. The same information was requested for all non-driver identification card holders. We specified the study’s DPPA exemption under permissible use Category 5. The request highlighted the fact that names were not needed to carry out the study and should be excluded from the database. The University of Vermont Committees on Human Research classified the study as the collection or study of existing data, waiving the requirement for individual consent.

Once contact was made with the appropriate government employee, phone and e-mail communications were used to further the data request. If at least three calls or e-mails were not returned, the state was labeled as “data unavailable due to no response.”

Each state had a unique process for releasing the data. Some required a signed memorandum of understanding outlining the specific uses for the data, the scope of our research, and technical systems in place for data security. Some sent the data without further paperwork. Some states were willing to send the data only with the address redacted to the zip code level. States also had various fees and waiting times. Each of these characteristics, along with the number of records, was recorded.

Data were read into Stata 14 (StataCorp LP, College Station, Texas) which was used to remove duplicate records (those with identical age, sex, and address) and calculate BMI and age. We considered the calculated BMI to be erroneous if height was less than 36 inches (91.4 cm) or more than 90 inches (229 cm), weight was less than 50 pounds (22.7 kg) or more than 599 pounds (271.7 kg), height was equal to weight, or calculated BMI was less than 8 or greater than 100 kg/m^2^. A set of regular expressions was used to identify post office boxes and other non-residential addresses. For some cases, the state of residence was inferred from the zip code. Where it could be inferred from other data, errors in the date of birth or date of issuance (usually due to errors in entering the century) were corrected. Age was calculated as the difference between the date of issue and the date of birth, expressed in years. Age was omitted if it was less than zero. Records were considered incomplete if they did not contain valid entries for age, sex, height, weight, calculated BMI, date of issue, or street address.

We summarized the availability of data by state and calculated the number of unique complete records as a proportion of the estimated state population in 2013 [17]. We calculated the fraction of complete records as the number of complete analyzable records divided by the total number of records provided by the state. We calculated the prevalence of obesity as the number of records with a BMI > 30 kg/m^2^ divided by the number of complete analyzable records. We assigned each record a 2010 Rural-Urban Commuting Area (RUCA) code derived from the zip code [18-19]. We divided the 10 RUCA codes into four categories representing Core Metropolitan areas (RUCA code 1), Outer Metropolitan areas (RUCA codes 2 and 3), Micropolitan areas, (RUCA codes 4-6), and Small Town and Rural areas (RUCA codes 7-10). For each category, we calculated the fraction of records that were normal or underweight, overweight, and obese.

We used chi-square tests to assess for statistical significance and logistic regression to adjust for the effects of age, sex, and RUCA codes on the prevalence of obesity.

## Results

All 50 states and the District of Columbia were contacted (Table 1). Nineteen states declined to provide any driver’s license data, citing state legislation preventing the release of protected information, departmental policy, and/or inadequate infrastructure to support such a request. Seven states do not record weight, making the calculation of BMI impossible. Four states indicated that data were available only with the address redacted to the zip code level. Six states and the District of Columbia either did not respond to multiple contacts or placed our request “under review,” but provided no follow-up response.

**Table 1:** Population by Response Category *Source: Annual Estimates of the Resident Population: April 1, 2010 to July 1, 2013 [17].

Response Category	Number of States	Population*	Population as % of US Population
*Total available for research (data includes address and weight)*	14	89,751,141	28.4%
Data received	7	62,082,353	19.6%
Data available, not acquired due to data fee	3	5,410,547	1.7%
Data approved, in queue for programming	3	10,687,433	3.4%
Data available for research, specific approval pending	1	11,570,808	3.7%
*Total data not available*	19	108,391,142	34.3%
Does not allow research use	13	81,142,793	25.7%
Does not provide/declined request /unable to process	6	27,248,349	8.6%
*Zip code only*	4	11,775,228	3.7%
*Weight not recorded*	7	74,097,334	23.4%
*No response*	7	32,113,994	10.2%
Total (including the District of Columbia)	51	312,513,753	100%

The remaining fourteen states indicated that driver’s license data with age, sex, height, weight, and full address were available for research. A total of 73.3 million driver’s license data records appear to be available, representing 82% of the population of these states and 23% of the 2013 population of the United States. The fees charged by the states range from no fee charged by multiple states to $30,000 (Nebraska) and even a quote of approximately $3,000,000 for Alaska’s 526,371 drivers. Individual state results are provided in Table 2.

**Table 2:** Data Availability by State *Source: Annual Estimates of the Resident Population: April 1, 2010 to July 1, 2013 [17].

State	Population*	Cost of Data (USD)	Street Address Available	Comments
Alabama	4,833,722		Unknown	No response
Alaska	735,132	$3M	No	Zip code only
Arizona	6,626,624		Unknown	No response
Arkansas	2,959,373	$4,000	Yes	Data not acquired due to data fee
California	38,332,521		No	Does not allow research use
Colorado	5,268,367		No	Does not allow research use (state law cited)
Connecticut	3,596,080		No	Weight not recorded
Delaware	646,449		No	Does not allow research use
District of Columbia	925,749		Unknown	No response
Florida	19,552,860		No	Weight not recorded
Georgia	9,992,167		Unknown	No response
Hawaii	1,404,054		No	Does not allow research use
Idaho	1,612,136		No	Declined data request
Illinois	12,882,135	$500	Yes	Data received
Indiana	6,570,902		No	Does not allow research use (state law cited)
Iowa	3,090,416	$0	Yes	In queue for data programming
Kansas	2,893,957		Unknown	No response
Kentucky	4,395,295		No	Declined data request
Louisiana	4,625,470		No	Declined data request
Maine	1,328,302	$95	Yes	Data received
Maryland	5,928,814		No	Does not allow research use
Massachusetts	6,692,824		No	Weight not recorded
Michigan	9,895,622	$0	Yes	Data received
Minnesota	5,420,380	$325	No	Zip code only
Mississippi	2,991,207		Unknown	No response
Missouri	6,044,171		No	Does not allow research use
Montana	1,015,165		No	Does not allow research use (state law cited)
Nebraska	1,868,516	$30,000	Yes	Data not acquired due to data fee
Nevada	2,790,136	$2,500	No	2-3 years to prepare data extract
New Hampshire	1,323,459		No	Does not allow research use (state law cited)
New Jersey	8,899,339		No	Does not allow research use. Also cited lack of “technical sophistication to fully accommodate” request.
New Mexico	2,085,287		No	Does not allow research use
New York	19,651,127		No	Weight not recorded
North Carolina	9,848,060		No	Weight not recorded
North Dakota	723,393		No	Does not allow research use (state law cited)
Ohio	11,570,808	$0	Yes	Data available for research, specific request under review
Oklahoma	3,850,568		Unknown	No response
Oregon	3,930,065	$292	Yes	Data received
Pennsylvania	12,773,801		No	Declined data request
Rhode Island	1,051,511		No	“Due to the age and fragility of our current legacy system…we are unable to provide” the data.
South Carolina	4,774,839	$0	No	Zip code only
South Dakota	844,877		No	Zip code only
Tennessee	6,495,978		No	Weight not recorded
Texas	26,448,193	$0	Yes	Data received
Utah	2,900,872		No	Does not allow research use
Vermont	626,630	$0	Yes	Data received
Virginia	8,260,405		No	Weight not recorded
Washington	6,971,406	$0	Yes	Data received
West Virginia	1,854,304	$300	Yes	In queue for data programming
Wisconsin	5,742,713	$0	Yes	In queue for data programming
Wyoming	582,658	$5,000	Yes	Data not acquired due to data fee
Total	316,128,839			

To date, we have received records of 53,794,943 unique individuals (Table 3) from seven states (Illinois, Maine, Michigan, Oregon, Texas, Vermont, and Washington). After excluding records without valid values for all the required fields, the dataset currently contains 52,621,861 analyzable records. Data files arrived as text files with standard delimiters. One state provided data on a password protected DVD; other states utilized FTPS to share their data. We anticipate receiving data from Iowa, Ohio, West Virginia, and Wisconsin with an estimated 15.5 million records. We have deferred purchases from Arkansas, Nebraska, and Wyoming (4.0 million estimated records) because of budgetary constraints, and we classified Nevada (1.7 million estimated records) as data unavailable because that state requires “two to three years” to prepare the data. An estimated 22.2 million additional records would be available if the seven states that did not respond to the request provide the data.

**Table 3:** Status of Available Data by State (n=14) *Source: Annual Estimates of the Resident Population: April 1, 2010 to July 1, 2013 [17].

State	Population*	Unique Persons in Datasets Received	Unique Analyzable Persons in Datasets Received	Analyzable Persons per 100 Population	Cost of Data (USD)	Street Address Data Available	Comments
Arkansas	2,959,373				$4,000	Yes	Data not requested due to data fee
Illinois	12,882,135	9,974,561	9,929,608	77.10%	$500	Yes	Data received
Iowa	3,090,416				$0	Yes	“In queue”
Maine	1,328,302	748,347	644,711	49%	$95	Yes	Data received
Michigan	9,895,622	9,429,739	8,634,217	87.30%	$0	Yes	Data received
Nebraska	1,868,516				$30,000	Yes	Data not requested due to data fee
Ohio	11,570,808				$0	Yes	“Under review”
Oregon	3,930,065	1,218,370	1,214,991	30.90%	$292	Yes	Data received
Texas	26,448,193	24,375,367	24,205,176	91.50%	$0	Yes	Data received
Vermont	626,630	561,285	552,706	88.20%	$0	Yes	Data received
Washington	6,971,406	7,487,274	7,440,452	106.70%	$0	Yes	Data received
West Virginia	1,854,304				$300	Yes	“In queue”
Wisconsin	5,742,713				$0	Yes	“In queue”
Wyoming	582,658				$5,000	Yes	Data not requested due to data fee
Total	89,751,141	53,794,943	52,621,861				

The seven states that have provided data represent New England, the South, the West and the Midwest. The records span a variety of time periods with the vast majority containing complete and valid values for age, sex, height, weight, and address suitable for geocoding and analysis (Table 4). There is a sizable heterogeneity, especially in age. Texas has a strikingly lower average age than the other states, a greater number of non-driver identification records, a very broad sample of historical data over 90 years, and the lowest proportion of females. BMI is highest, and obesity most prevalent, in Michigan. They are lowest in Washington state.

**Table 4:** Driver Characteristics by State of Issuance BMI = Body Mass Index in kg/m^2^

State	Years of Issuance	Complete Records	Age, Mean (range)	Male	BMI, Mean (range)	Obese
Illinois	1969-2014	99.5%	43.7 (0-110)	50.1%	26.0 (8.1-99.7)	18.9%
Maine	1975-2014	86.2%	46.1 (0-106)	49.6%	25.9 (10.5-85.8)	17.8%
Michigan	1915-2014	91.6%	33.2 (0-100)	50.7%	27.0 (8.1-99.8)	25.1%
Oregon	1996-2014	99.7%	57.6 (0-107)	51.2%	26.4 (8.6-94.8)	20.4%
Texas	1916-2014	99.3%	25.8 (0-108)	51.1%	26.7 (8.0-99.8)	23.6%
Vermont	2000-2014	98.5%	45.6 (14.2-103)	49.6%	25.9 (8.3-91.8)	17.7%
Washington	1964-2013	99.4%	40.8 (0-120)	50.3%	25.5 (8.1-99.4)	15.8%
Total	1915-2014	97.8%	33.8 (0-120)	50.7%	26.4 (8.0-99.8)	21.7%

**Figure 1:**
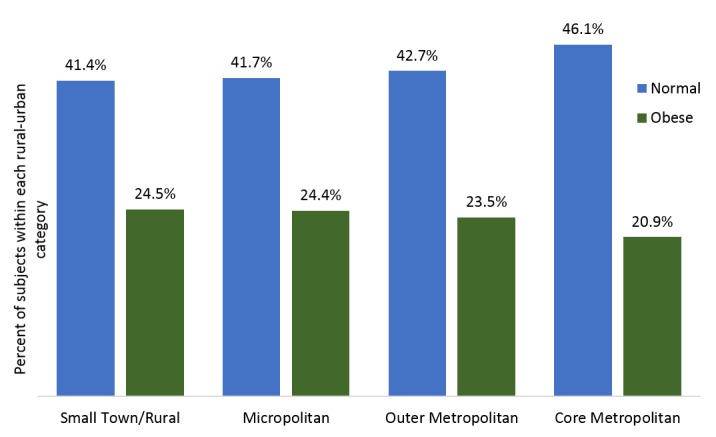
Unadjusted distribution of normal and obese body mass across the rural-urban development spectrum

Obesity was more common in males than in females (22.3% *vs.* 21.0%; *P *< 0.001) and varied with age among adults (20-39 years: 20.2%; 40-49 years: 23.9%; > 60 years: 23.1%; *P *< 0.001). The prevalence of obesity varied monotonically with the position of the address on the Rural-Urban spectrum. Obesity was most common in Rural and Small Town areas and least common in Core Metropolitan areas (Figure 1). Likewise, the prevalence of normal and underweight subjects fell as the degree of urbanization declined (all differences significant with *P *< 0.001).

The range of obesity rates across the seven states was 9.3% (15.8% to 25.1%). However, after adjustment for differences among the states in age, sex, years of issue, and rural-urban status, the range fell to 2.3% (20.1% to 22.4%). In other words, much of the variation in the distribution of obesity can be attributed to personal and environmental factors rather than systematic differences among the states in their data collection systems.

Incomplete records numbered 1,173,082 (2.2% of all unique records) and tended to have more recent dates of issue (mean: September 2002 vs. April 2001; *P *< 0.001). Subjects with incomplete records were younger (33.7 vs. 33.8 years; *P *= 0.016), more often male (54.2% vs.50.6%; *P *< 0.001), had greater BMIs (26.5 vs. 26.4 kg/m^2^; *P *< 0.001), and were more often obese (23.0% vs. 21.6%; *P *< 0.001) than those with complete records.

## Discussion

State driver’s license data may provide a large source of valuable data for epidemiological research, particular for studies of obesity. However, the personal information that makes these databases attractive to researchers are also the reason driver’s license records are considered restricted information. The federal DPPA was signed into law in 1994 in order to combat abuse of drivers’ personal information [15]. Following the DPPA, many states enacted their own legislation that further reduced the availability of driver’s license data. However, the inclusion of the DPPA’s Exemption Category 5 currently provides researchers access to over 50 million records from all parts of the country with a broad representation of all urban and rural geographies and demographic subgroups.

The availability of state driver’s data is subject to change, as demonstrated when comparing this study and that of Walsh, et al. [16]. During our research, we were approved to receive the driver’s license data from six states (Arkansas, Illinois, Iowa, Oregon, Washington, and Wyoming) previously found to have restricted data. However, Walsh, et al. were able to gain approval from Utah, which declined our request. The discrepancies between the two findings may be a result of changing state legislation or shifting departmental policies within the organizations that administer driver’s license data. Additionally, some states may have responded differently to our request, as we submitted an actual research data request, rather than a hypothetical request as submitted by Walsh, et al.

Only two states (New Jersey and Rhode Island) cited the inability to produce the requested report. Rhode Island reported that it is in the process of implementing a new driver’s license database system that will be able to handle research data requests. Five states (Colorado, Indiana, Montana, New Hampshire, and North Dakota) declined data requests based solely on state legislation. Therefore, it appears that the remaining states have the technical capability to produce requested driver’s license data reports and are not bound by state law to deny research requests. As departmental policies change, additional data sets may become available.

States vary in the number of records as a proportion of their total population. The data from Oregon covered less than one-third of the state’s population while Washington provided more records than their entire population. States may vary in eligibility for licenses (especially for teenaged drivers and felons) and non-driver identification cards (especially for undocumented immigrants), how thoroughly they purge the licenses of former and deceased residents, the prevalence of fraudulent duplicative licenses, the proportion of out-of-state residents with local licenses (retirees, college students, military personnel, etc.), and the completeness of the data extracts they sent us. The variability in age and sex across the states is, at this time, largely unexplained, although these administrative differences may be responsible.

It is impossible to confirm if any licensed drivers or holders of non-driver identity cards were omitted by the state agencies. However, the available data appear to be remarkably complete, with only 2.2% of records missing any of age, sex, height, weight, address, or year of issue. All of the states that contributed data require all of the elements we report on, but they may vary in how strictly they enforce this requirement. Although the incomplete records are statistically significantly different than the complete records in those characteristics, the differences are generally quite small. Given a difference of 0.1 years of age and 0.1 kg/m^2^ of BMI, it seems unlikely that the incomplete records represent a population that is importantly different than the complete records.

BMI calculated from driver’s license data have several important limitations. The data are not strictly current but represent the driver’s report at the time of issuance, which can be very many years ago. In some cases, it is unclear if the height and weight were updated at the latest date of issuance, or represent earlier data that were carried forward from a prior issuance. The data are subject to all the vagaries of administrative information, including empty fields, physiologically impossible heights, weights, and ages, and missing or uninterpretable addresses. Some addresses, such as post office boxes, do not represent residences. There is usually no information on how long the driver resided at the address.

Importantly, the data derive from self-report of height and weight with little, if any, validation. Almost certainly, drivers tend to underestimate weight and overestimate height resulting in systematic underestimates of BMI. For instance, the driver’s license data suggest a prevalence of adult obesity of 21.9%, compared to 34.3% when measured directly [20]. This bias makes the data unsuitable for estimating the absolute value of BMI or prevalence of obesity. However, the data retain utility for analyzing relationships between geographic factors and obesity if the error in BMI is not correlated with the place. For instance, if the tendency to underestimate BMI is similar in rural and urban areas, then the relative difference in obesity in these areas can be estimated without bias. Indeed, BMI and obesity calculated from driver’s license data vary as previously reported with the rural-urban development gradient [21]. 

In spite of these limitations, driver’s license data have many strengths. Given that the US Census has never included reports of height and weight, they may provide the most complete population of adults available for the study of obesity, its relationship to local policies, and natural and built features of the environment in the United States. Even without further details, such as ethnicity, personal habits, and economic factors, this large and broadly applicable data set provides advantages over more labor-intensive methods.

## Conclusions

Although driver’s license data are restricted information with important limitations, public health researchers can gain access to tens of millions of valuable records. Given the dearth of other large datasets with specific locations as well as health information, driver’s licenses represent an underused resource for studying the geographic correlates of obesity and other public health issues.
